# Improving the Diagnosis of Dermatitis Herpetiformis Using the Intraepithelial Lymphogram

**DOI:** 10.3390/nu16020232

**Published:** 2024-01-11

**Authors:** Fernando Fernández-Bañares, Laura Crespo, Montserrat Planella, Sergio Farrais, Sandra Izquierdo, Natalia López-Palacios, Garbiñe Roy, Judith Vidal, Concepción Núñez

**Affiliations:** 1 Department of Gastroenterology, Hospital Universitari Mutua Terrassa, 08221 Terrasa, Spain; ffbanares@mutuaterrassa.es; 2 Centro de Investigación Biomédica en Red de Enfermedades Hepáticas y Digestivas (CIBERehd), Instituto de Salud Carlos III, 28029 Madrid, Spain; 3 Department of Gastroenterology, Hospital Universitario Ramón y Cajal, 28034 Madrid, Spain; lcreper@yahoo.es; 4 Department of Gastroenterology, Hospital Arnau Vilanova, 25198 Lleida, Spain; mplanella.lleida.ics@gencat.cat; 5 Department of Gastroenterology, Hospital Fundación Jiménez Díaz, 28040 Madrid, Spain; sfarraisv@quironsalud.es; 6 Department of Gastroenterology, Hospital Clínico Universitario, 47003 Valladolid, Spain; sizquierdos@saludcastillayleon.es; 7 Department of Gastroenterology, Hospital Clínico San Carlos, Instituto de Investigación Sanitaria Hospital Clínico San Carlos, 28040 Madrid, Spain; natalia.lopa@gmail.com; 8 Department of Immunology, Hospital Universitario Ramón y Cajal, Instituto Ramón y Cajal de Investigación Sanitaria, 28034 Madrid, Spain; garbine.roy@salud.madrid.org; 9 Section of Flow Cytometry, CATLAB, 08232 Viladecavalls, Spain; jvidal@catlab.cat; 10 Laboratorio de Investigación en Genética de Enfermedades Complejas, Hospital Clínico San Carlos, Instituto de Investigación Sanitaria Hospital Clínico San Carlos, 28040 Madrid, Spain; 11 Redes de Investigación Cooperativa Orientada a Resultados en Salud (RICORS), 28029 Madrid, Spain

**Keywords:** dermatitis herpetiformis, celiac disease, celiac intraepithelial lymphogram, T-cell flow cytometry, gluten-free diet, TCRγδ^+^ cells

## Abstract

Dermatitis herpetiformis is a cutaneous manifestation of celiac disease. Phenotyping of intraepithelial lymphocytes in the small bowel mucosa can strengthen the diagnosis of celiac disease when it is not clear-cut. We aim to evaluate the usefulness of the intraepithelial lymphogram to confirm dermatitis herpetiformis in equivocal cases. We performed a retrospective multicenter study on patients diagnosed with dermatitis herpetiformis and collected data from the intraepithelial lymphogram assessed by flow cytometry. A total of 36 patients were analyzed in relation to the severity of intestinal damage (18 had non-atrophic mucosa) at baseline (N = 28) and/or after the adoption of a gluten-free diet (median follow-up of three years, N = 16). We observed that patients with atrophy more often had positive celiac serology (*p* = 0.019), celiac clinical symptoms (*p* = 0.018), and iron-deficiency anemia (*p* = 0.018), but the severity of skin damage was similar in both groups (*p* = 0.79). At baseline, increased TCRγδ^+^ cells were present in 94% of patients with atrophy and 67% with non-atrophic lesions (*p* = 0.13). After a gluten-free diet, increased TCRγδ^+^ cells persisted in 100% and 63% of cases, respectively (*p* = 0.21). We concluded that increased TCRγδ^+^ cells may be helpful in confirming the diagnosis of dermatitis herpetiformis in equivocal cases, even in patients who were started on a gluten-free diet.

## 1. Introduction

Dermatitis herpetiformis (DH) is a cutaneous manifestation of celiac disease (CD) presenting with intense itching and a symmetrical blistering rash, typically seen on the elbows, knees, and buttocks [1,2,3]. Although overt gastrointestinal symptoms are rare, approximately 70% of patients with DH have villous atrophy, and the remainder have potential CD-like inflammatory changes [1]. Currently, the prevalence of DH to CD is 1:8. The incidence of DH is decreasing, while that of CD is increasing, probably due to earlier diagnosis [3]. The diagnosis of DH is confirmed by the observation of granular deposits of immunoglobulin A in the papillary dermis by direct immunofluorescence (DIF). A biopsy of normal-appearing skin adjacent to the rash, i.e., perilesional skin, should be taken because IgA deposits predominate near active lesions. False-negative DIF results may occur in approximately 5% of patients [1,2,3,4] and are more common if the biopsy is taken from blisters or inflamed skin. Additionally, technical errors, failure of current laboratory methods to detect cutaneous IgA deposits in some patients, and focal deposition of IgA in the skin may explain the negative DIF in DH [5]. In addition, patients with a rash resembling DH, the deposition of exclusively complement (C3) at the dermal-epidermal junction, and response to a gluten-free diet (GFD) have been described [6]. These patients may represent a new disease entity, possibly related to non-celiac gluten sensitivity, and emphasize that the response to a GFD cannot be used as the sole criterion for the diagnosis of CD. 

By definition, CD is excluded in patients with non-atrophic small intestinal mucosal morphology who follow a normal gluten-containing diet. However, it seems evident that this is not accurate since clinical manifestations may develop in the early stages of the disease when the mucosa is still morphologically “normal” [7,8]. Today, such patients are often referred to as having potential CD and are not always treated as celiacs with a GFD [9], although this could lead to clinical remission [7,8,10,11,12,13,14]. In this sense, up to 30% of DH patients present with the absence of intestinal mucosal villous atrophy, with celiac serology being negative in 60% of them [15]. In addition, they have increased intraepithelial TCRγδ^+^ cells [8].

Nowadays, routine intestinal biopsies are not necessary when the diagnosis of DH is straightforward [1,2,3]. This is the case with typical results of DIF and positive celiac serology [16,17]. However, duodenal biopsies may be helpful either in equivocal cases with a skin rash typical of DH but negative DIF [2], or in cases with positive DIF but negative celiac serology [16]. In patients with negative DIF, new skin biopsies may be taken. However, in some patients with confirmed DH, even repeated biopsies taken at different times have yielded negative DIF results [18]. In these cases, the presence of the characteristic distribution of intraepithelial lymphocytes (IELs) observed in CD may be a clue to the diagnosis of DH, i.e., patients with villous atrophy but negative celiac serology [19], and those with non-atrophic Marsh type 1 lesions, in whom celiac serology is often negative [20,21]. In the search for markers of early CD, intraepithelial TCRγδ^+^ cells have been shown to increase in both atrophic and non-atrophic CD lesions, even in patients with negative celiac serology [7,19,22].

Several studies have shown that TCRγδ^+^ cells in the small bowel mucosa are similarly increased in patients with DH and in patients with CD [23,24,25]. In most cases, neither long-term adherence to a GFD nor the morphology of the small intestine influenced the density of TCRγδ^+^ cells [23]. These landmark studies were performed using immunohistochemistry techniques to assess the density of TCRγδ^+^ cells. The TCRγδ^+^ subset could be quantified more accurately with the introduction of flow cytometry [26,27,28,29]. The combined use of a high percentage of TCRγδ^+^ and a low percentage of CD3^−^ cells, which was termed the celiac lymphogram, was shown to be highly accurate for CD diagnosis, even in patients with Marsh 1 lesions [22,29,30]. The isolated increase in TCRγδ^+^ cells was also associated with CD diagnosis. In a meta-analysis of published studies, the celiac lymphogram pattern had a pooled sensitivity and specificity of 93% and 98% for CD diagnosis, while only considering the increase in TCRγδ^+^ cells (with or without the concomitant decrease in CD3^−^ cells), the sensitivity and specificity were both 95% [31]. Thus, the phenotyping of IELs by flow cytometry constitutes a highly sensitive and specific complement to serology and histological examination for the diagnosis of CD, even in individuals with CD and negative celiac serology or in those following a GFD who exhibit normal duodenal histology [29]. In addition, the celiac lymphogram pattern is absent in patients with clinical conditions other than CD [27,30].

In conclusion, non-atrophic mucosa is common in DH and often presents in the absence of other accompanying symptoms/signs, and with negative celiac serology [32]. This may make the diagnosis of DH difficult when it is not straightforward. Considering that there are no differences in the clinical cutaneous picture and response to a GFD between patients with DH showing atrophic or non-atrophic changes, new tools to assist in the diagnosis of celiac disease and, by extension, the diagnosis of DH are needed. Thus, the main aim of the present study was to investigate the presence of the celiac lymphogram in patients with DH in order to evaluate its usefulness in the diagnosis of equivocal cases of DH as a complement to celiac serology and histopathological examination. In addition, we consider the following two secondary endpoints: (1) to compare the main CD-related characteristics in DH patients with atrophic and non-atrophic duodenal mucosa; (2) to evaluate the usefulness of the IEL lymphogram specifically in DH patients who have already started a GFD.

## 2. Materials and Methods

### 2.1. Study Design

This was a retrospective multicenter study searching the records of the anatomical pathology departments of the 6 participating centers (Hospital Universitari Mutua Terrassa, Barcelona; Hospital Arnau Vilanova, Lleida; Hospital Clínico Universitario, Valladolid; Hospital Universitario Ramón y Cajal, Hospital Fundación Jiménez Díaz, and Hospital Clínico San Carlos, Madrid) for all patients diagnosed with DH in the period from January 2000 to September 2023. Inclusion criteria for the list of patients on record were as follows: (1) diagnosis of DH based on the typical cutaneous picture and the presence of granular deposits of immunoglobulin A in the papillary dermis by direct immunofluorescence, and (2) baseline intestinal biopsies on a normal gluten-containing diet. This study was focused on the patients who had IEL lymphogram determination by T-cell flow cytometry at baseline and/or the follow-up biopsy. Therefore, some centers included patients recruited after 2000, when they started to have IEL lymphogram data available. Patient recruitment in all centers ended in September 2023.

Demographic and clinical variables, celiac serology, HLA-DQ-phenotype, response to GFD, and associated treatments (dapsone) were registered. Skin lesions were classified as mild, moderate, or severe based on the presence of few, several, or multiple blisters/vesicles, macular rash, or erosions [15]. After diagnosis, a strict GFD was recommended to all patients, and dapsone was introduced in those with severe skin symptoms. Response to a GFD was considered a diagnostic criterion for DH, as was early response to dapsone treatment in patients who required it [33].

Seronegative patients showing a Marsh 1 lesion were considered to have low-grade celiac enteropathy if there were any of the following: 1. Exclusion of all the other causes of a Marsh 1 lesion, like the absence of concomitant diseases associated with immune dysregulation, Helicobacter pylori, parasitosis, and not taking non-steroidal anti-inflammatory drugs nor olmesartan. 2. A permissive HLA-DQ genotype. 3. Presence of the celiac lymphogram by flow cytometry. 4. Sustained clinical remission and a histological response to a GFD. Thus, the celiac lymphogram was used as an additional criterion for seronegative Marsh 1 patients with ‘low grade celiac enteropathy’ [7,34]. 

### 2.2. Duodenal Biopsy Assessment for Histopathology

Four biopsies were taken from the second portion of the duodenum and one from the duodenal bulb in patients who underwent an esophagogastroduodenoscopy. They were processed using hematoxylin/eosin staining and CD3 immunophenotyping. CD Marsh 3 damage was defined as villous atrophy, crypt hyperplasia, and an increased IEL count. Marsh 1 lesions (lymphocytic enteritis) were defined by more than 25 IELs per 100 epithelial cells along with normal villous architecture. The IEL count was performed as previously described [35]. 

### 2.3. Celiac Serology

Serum IgA tissue anti-transglutaminase antibodies (tTG) (or IgG tTG in IgA-deficient patients) were analyzed using homologated commercial quantitative automated ELISAs while the patients were on a gluten-containing diet at the time of the diagnosis (during the 23 years considered for patient recruitment). Depending on the center, Elia Celikey IgA (Thermo Fisher, Phadia AB, Uppsala, Sweden), Aeskulisa tTg-A (Aesku Diagnostics, Wendelsheim, Germany), or Bioplex 2200 Celiac IgA (Bio-Rad, Hercules, CA, USA) were mainly used. Little change in sensitivity was expected. Serum anti-endomysium IgA antibodies (EmA) were tested by an IF assay at a dilution of 1:5 in commercial sections of primate distal esophagus as the antigen substrate (Immco Diagnostics, Buffalo, NY, USA) to confirm a positive result in all samples analyzed with the Aeskulisa tTG-A kit. In the remaining centers, EmA was tested in patients with either borderline anti-tTG or detectable anti-tTG titers that were below the cut-off suggested by the manufacturer to confirm positive celiac serology.

### 2.4. Flow Cytometry Analysis

As part of the initial diagnostic process, one duodenal biopsy from the second–third portion of the duodenum was obtained and processed immediately for IEL flow cytometry, as previously described [9,23]. All centers used similar gating strategies to select both CD3^−^ and TCRγδ^+^ cells, consisting of quantifying CD45^+^ CD103^+^ CD3^−^ and CD45^+^ CD103^+^ TCRγδ^+^ cells, respectively, over the total of CD45^+^ CD103^+^ cells. Comparative studies on samples analyzed in parallel in different hospitals showed a concordance of almost 100% in terms of absolute percentages for both CD3^−^ and TCRγδ^+^ cells [36].

**Figure 1:**
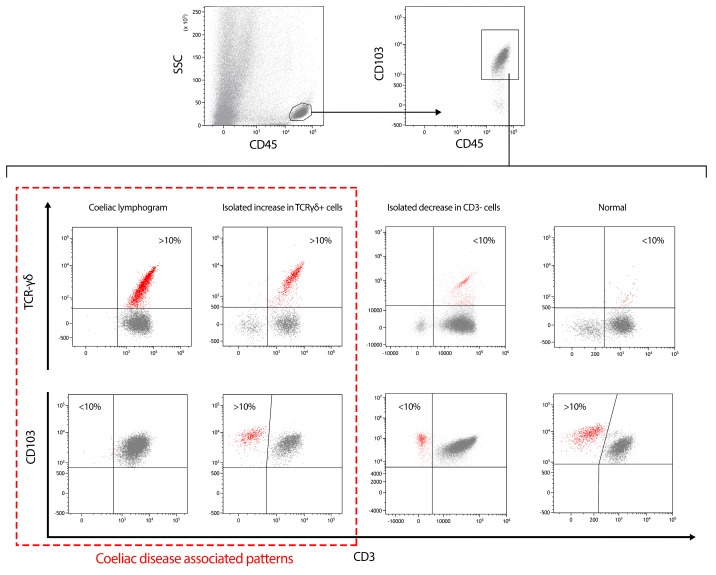
Gating strategy used for the study of the intraepithelial lymphogram followed by the four possible patterns found. The two patterns associated with celiac disease diagnosis (celiac lymphogram and isolated increase in TCRγδ^+^ cells patterns) are in a dashed red square.

The different participating centers routinely used diverse cut-offs, ranging from >8.5 to 12%, with most centers using >10% for TCRγδ^+^ cells and <10% for CD3^−^ cells, which entails slight variations in sensitivity and specificity between the centers. It must be considered that the lower the cut-off, the higher the sensitivity, and vice versa. Some centers consider specificity to be more relevant, and others consider sensitivity more important. For this study, we preferred to extract the recorded individual quantitative values of TCRγδ^+^ and CD3^−^ cell percentages and interpret them with a single cut-off value that allows for a high specificity while maintaining good sensitivity. Thus, the celiac lymphogram was defined as a >10% increase in TCRγδ^+^ cells plus a concomitant <10% decrease in CD3^−^ cells. These cut-offs define four intraepithelial lymphogram patterns: normal, isolated decrease in CD3^−^ cells, isolated increase in TCRγδ^+^ cells, and the celiac lymphogram. The latter two patterns showing increased TCRγδ^+^ cells (with or without the concomitant decrease in CD3^−^ cells) are associated with CD. The gating strategy and the four patterns are illustrated in Figure 1.

Around 85–93% of CD patients showed a celiac lymphogram, 2–14% showed an isolated increase in TCRγδ^+^ cells, and 3–8% showed a non-celiac pattern (either an isolated decrease in CD3^−^ cells or a normal pattern) [30,31]. The increase in TCRγδ^+^ cells (celiac lymphogram plus isolated increase in TCRγδ^+^ cells) is observed in 93–97% of CD patients. 

### 2.5. Statistical Analysis

The results are expressed as the mean ± standard deviation, median, and interquartile range (IQR) or percentages. The chi-square test, Fisher’s exact test and the Freeman–Halton extension of the Fisher exact probability test for a 2 × 3 contingency table were used for qualitative variable comparisons. Student’s *t* test or the Mann–Whitney U test was used for quantitative variables when appropriate. The Kolmogorov–Smirnov test was assessed to determine whether data were normally distributed. Statistical significance was set at *p* < 0.05. Analyses were performed using SPSS (IBM Corp. Released 2010. IBM SPSS Statistics for Windows, Version 19.0. Armonk, NY, USA). 

## 3. Results

**Figure 2:**
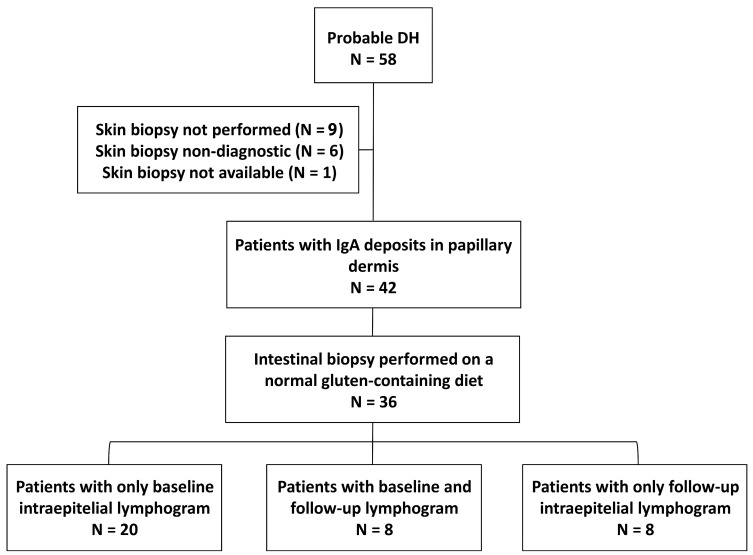
Flow chart of the study. From the initial database, 36 patients were finally studied because they had cutaneous IgA deposits and a duodenal biopsy performed; they were grouped depending on the presence of baseline (N = 28) or follow-up (N = 16) intraepithelial lymphograms. Eight patients were included in both groups.

The flow chart of the study is shown in Figure 2. A total of 57 patients were analyzed by a cutaneous picture suggestive of DH, but IgA granular deposits in the papillary dermis could only be demonstrated in 42 patients. Of these, small intestinal biopsies were obtained in 36 patients on a gluten-containing diet and were included in the study. The intraepithelial lymphogram was assessed at baseline in 28 patients and at follow-up while on a GFD in 16 patients (8 were analyzed both at baseline and follow-up).

Of the 36 included patients (50% male, age 43.7 ± 3.1 years), 50% had non-atrophic lesions (N = 18; 8 Marsh 0, which corresponds to histologically normal small bowel mucosa, with unaltered villous architecture and less than 25–30 IELs per 100 epithelial cells; 9 Marsh 1; and 1 Marsh 2) and the other 50% had atrophic lesions (N = 18). Table 1 describes the comparison of the study variables between these two groups. Patients with atrophic lesions were more likely than those with non-atrophic lesions to have positive serology, clinical symptoms on the spectrum of CD, and iron deficiency anemia; however, the severity of the cutaneous picture was similar between the two groups. The clinical response to a GFD was 100% in both groups. A total of 22.2% of non-atrophic lesions versus 38.9% of atrophic lesions required the additional use of dapsone medication (*p* = 0.23). Serologic response (negative anti-tTG after GFD) was 100% and 94% in the non-atrophic and atrophic groups, respectively.

### Intraepithelial Lymphogram

**Figure 3:**
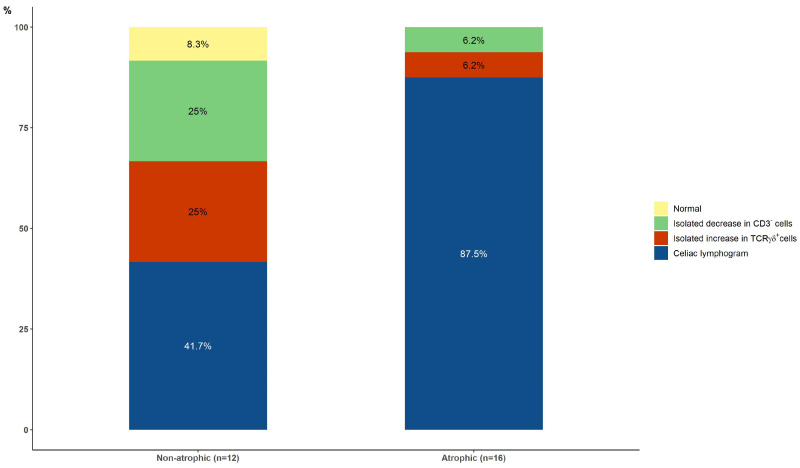
Baseline intraepithelial lymphograms in patients with non-atrophic and atrophic lesions (*p* = 0.010, celiac lymphogram vs. others).

At baseline, the celiac lymphogram was present in 5/12 (41.7%) patients with non-atrophic lesions and in 14/16 (87.5%) patients with atrophic lesions (*p* = 0.017) (Figure 3). 

**Figure 4:**
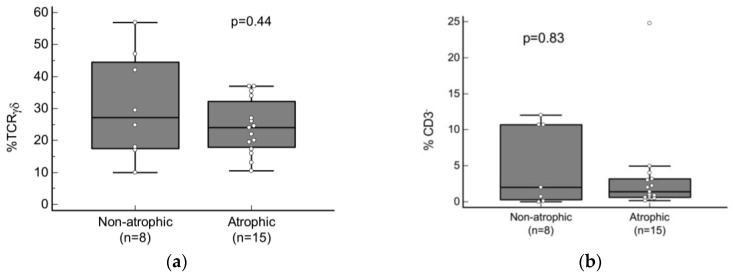
Comparison of median baseline count of (**a**) TCRγδ^+^ (CD45^+^ CD103^+^ TCRγδ^+^/CD45^+^ CD103^+^) and (**b**) CD3^−^ (CD45^+^ CD103^+^ CD3^−^/CD45^+^ CD103^+^) cells between patients with non-atrophic and atrophic lesions who presented with one of the two cytometric patterns associated with CD (the celiac lymphogram or the isolated increase in TCRγδ^+^ cells patterns). *p* values were calculated using a Mann–Whitney U test.

One of the two cytometric patterns associated with CD (the celiac lymphogram or the isolated increase in TCRγδ^+^ cell patterns) was present in 15/16 (93.7%) patients with atrophic lesions and in 8/12 (66.7%) patients with non-atrophic damage (*p* = 0.13). There were no significant differences in the median count of either TCRγδ^+^ or CD3^−^ cells in patients with a cytometric pattern associated with CD comparing atrophic and non-atrophic lesions (Figure 4a,b).

Considering the sample size of 28 subjects and the observed percentage of 82% of increased TCRγδ^+^ cells, our study has a precision of ±14% with a 95% confidence level. 

**Figure 5:**
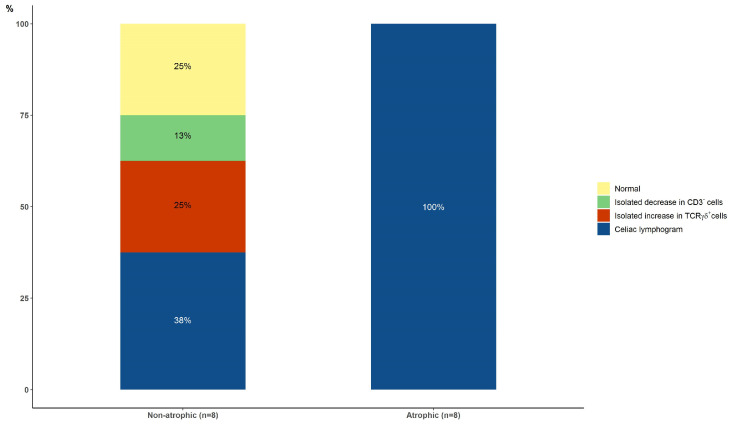
Follow-up intraepithelial lymphograms after adopting a gluten-free diet in patients with initial non-atrophic and atrophic lesions.

Only one out of the sixteen patients with a follow-up intraepithelial lymphogram had persistent atrophy at the follow-up biopsy. Four patients had Marsh 1 lesions, and eleven patients had Marsh 0 lesions. The results after a median follow-up on a GFD of 3 years (IQR, 3 to 12) showed that an increase in TCRγδ^+^ (with or without a concomitant decrease in CD3^−^ cells) was present in 62.5% and 100% of the initial non-atrophic and atrophic groups, respectively (*p* = 0.21) (Figure 5).

Regarding celiac serology, 20 out of 23 seropositive patients (16 Marsh 3, 4 Marsh 1, and 3 Marsh 0) had an increase in TCRγδ^+^ cells (90% with the celiac lymphogram), and the other 3 had an isolated decrease in CD3^−^ cells (1 Marsh 3 and 2 Marsh 1). In addition, three out of the five patients with negative serology (4 Marsh 0 and 1 Marsh 1) had an increase in TCRγδ^+^ cells (20% with the celiac lymphogram, *p* = 0.025 vs. seropositive patients) and one had an isolated decrease in CD3^−^ cells. Only two out of the twelve patients with non-atrophic lesions (17%) were seronegative and had a normal cytometric pattern.

## 4. Discussion

The present study in six Spanish University Hospitals with expertise in CD allowed the selection of 36 consecutive patients with DH in whom the intraepithelial lymphogram had been assessed at baseline and/or at a median 3-year follow-up with a GFD. The results provide new information on the usefulness of this assay to support the diagnosis of DH in patients with uncertain diagnoses, even if they have started a GFD.

A total of 50% of the included patients had non-atrophic lesions (Marsh type 0 to 2 lesions), which is higher than the 25% reported in a previous study in a very large sample of Finnish patients over a time span of almost 50 years [37]. In that study, the diagnosis of DH before the year 2000 was significantly associated with a longer delay in diagnosis, so it is possible that initial mild intestinal damage had evolved into atrophic lesions by the time of DH diagnosis. The authors concluded that small bowel mucosal injury had become less severe in DH in recent decades, perhaps due to an earlier diagnosis.

We also observed that patients with non-atrophic enteropathy presented less frequently with celiac symptoms and signs other than the cutaneous picture compared to patients with atrophic lesions. This contrasts with a previous study that showed that the presence of villous atrophy was not associated with the occurrence of gastrointestinal symptoms at diagnosis [15]. However, data on clinical symptoms were collected by questionnaires after a long-time lapse, so there is a possibility of recall bias. The percentage of seronegative disease in the present series was 17%, and in the literature, it has ranged from 8% to 40% [15,38,39]. In our study, seronegative disease was more frequent in patients with non-atrophic lesions.

DH may occur even with a normal small bowel mucosa [1,3], but in most cases with non-atrophic lesions, there is celiac-type inflammation and/or immune response. As mentioned above, both DH and CD are characterized by increased densities of TCRγδ^+^ IELs in the small bowel mucosa; however, as shown in the present series, there is a small percentage of patients who do not present this typical celiac-like inflammation. The increase in TCRγδ^+^ cells had a sensitivity of 94% for patients with villous atrophy, which is a figure similar to previous studies [30,31]. One elderly patient had an isolated decrease in CD3^−^ IELs, which is a pattern observed in some CD patients who have not developed an increase in the γδ+ subset despite the presence of villous atrophy, mainly observed in children and the elderly [30,36]. Follow-up biopsies in the group with villous atrophy showed that the increase in TCRγδ^+^ cells persisted over time, despite the adoption of a GFD and normalization of histopathology. In patients with non-atrophic lesions, the results were not so good, and an increase in TCRγδ^+^ IELs was present in nearly 70% of patients at diagnosis and also persisted over time. 

As the presence of an intraepithelial increase in TCRγδ^+^ cells favors the presence of CD, a routine evaluation of the intraepithelial lymphogram may be useful to confirm the characteristic celiac IEL distribution in those patients with probable DH, in whom the diagnosis is unclear and duodenal biopsies for histopathology are indicated. The presence of non-atrophic mucosa is common in DH, as shown in the present series, and in these cases, a correct diagnosis of a gluten-related entity is not easy to perform without the aid of the intraepithelial lymphogram assessment. The current results in DH patients are consistent with previous studies in seronegative CD [19] or in low-grade celiac enteropathy [7,22,34,36], showing that the intraepithelial lymphogram assay is highly accurate in predicting CD. Seronegative CD is present in a reduced percentage of patients with the disease, and non-atrophic seronegative CD is considered even rarer. The high impact on the quality of life of these patients when starting a GFD after diagnosis underlines the importance of a correct diagnosis. Since both situations, seronegative disease and non-atrophic mucosa, can be found in DH patients, and considering that negative DIF results are observed in some DH patients, as mentioned in the Introduction, the diagnosis of DH and CD in some cases requires a careful evaluation of each individual patient, and the celiac lymphogram can be a very useful tool.

CD diagnosis is also challenging for patients who have started a GFD. In fact, some patients with a previous diagnosis of DH whose rash resolves after a GFD come to the clinic with no information on how the diagnosis was made. A new skin biopsy showing a positive DIF, since subdermal IgA deposits can persist for a long time despite a GFD [40], would confirm the diagnosis of DH. However, DIF-negative results could also be obtained despite DH. In this scenario, the increase in TCRγδ^+^ cells could allow detection of celiac enteropathy and the need to continue a strict lifelong GFD. In addition, it would rule out non-celiac gluten sensitivity, in which there is no increase in intraepithelial TCRγδ^+^ cells [41].

In general, the assessment of the density of TCRγδ^+^ IELs has been performed with immunohistochemistry techniques in frozen small bowel biopsy samples. However, conclusions may be difficult to draw because it is a user-dependent and laborious technique that requires well-oriented and high-quality samples. In fact, its use has been limited to research purposes and has not been extended to clinical practice. The present study is the first to use flow cytometry to quantify TCRγδ^+^ IELs in DH. The assessment of TCRγδ^+^ IELs by flow cytometry allows for accurate quantification and is a highly reproducible methodology applicable in routine clinical practice [29]. In fact, flow cytometry represents a simple, fast, and inexpensive tool for diagnosis.

The main limitation of the present study is the small sample size. However, DH is a rare disease with a decreasing incidence and a prevalence of 1–7.5/10,000 persons [42,43], but prevalence data in most European countries are unknown. Most of the clinical data come from Finnish studies, where the disease is more common, and in fact, the number of published series of patients with DH is very small, as it is difficult to collect a large sample of patients with the disease. The present study provides data on a new approach to diagnosing DH in equivocal cases. Although based on 36 patients (despite the participation of six hospitals), our results warrant further studies using a flow cytometry assay to assess the intraepithelial lymphogram for diagnostic purposes. A second limitation of the study is that it is a multicenter study utilizing different flow cytometric analysis/thresholds, which may lead to some degree of heterogeneity; however, we performed comparative studies showing a high degree of concordance between centers.

## 5. Conclusions

Although the sample size is small, the intraepithelial lymphogram represents a useful parameter that supports a subjacent celiac process in equivocal cases of DH, even in those with minimal intestinal lesions and poor antibody responses or when a GFD has already been initiated. The increase in TCRγδ cells (an almost pathognomonic finding in CD) is present in 94% of DH patients with villous atrophy and around 70% of those with non-atrophic damage. 

A new study, with a larger sample size and preferably prospective participants, is needed to corroborate our findings and validate the true utility of this tool in relation to DH.

## Figures and Tables

**Table 1:** Comparison of study variables between patients with atrophic and non-atrophic intestinal lesions.

Variable	Atrophic Lesions (N = 18)	Non-Atrophic Lesions (N = 18)	*p* Value
Age (years)	42.9 ± 4.4	44.6 ± 4.4	0.80
Sex (% male)	8 (44.4%)	10 (55.5%)	0.50
Severity of the cutaneous lesion			
Mild	3 (16.7%)	5 (27.7%)	0.79
Moderate	12 (66.7%)	11 (61.1%)	
Severe	3 (16.7%)	2 (11.1%)	
Symptoms of CD			
Symptom-free	9 (50%)	16 (88.9%)	0.034
Diarrhea and/or abdominal pain	7 (38.9%)	2 (11.1%)	
Other	2 (11.1%)	0%	
Presence of IDA *	8 (44.4%)	1 (5.5%)	0.018
Positive celiac serology **	18 (100%)	12 (66.7%)	0.019
HLA-DQ genotyping			
HLA-DQ2.5	15 (83.3%)	16 (88.9%)	1
HLA-DQ8	2 (11.1%)	2 (11.1%)	
HLA-DQ2.2	1 (5.5%)	0%	

* IDA: Iron-deficiency anemia was established based on hemoglobin, MCV and ferritin values. ** See Materials and Methods for details.

## Data Availability

The database on which this article is based is available upon reasonable request.
